# 

**Published:** 1908-10-31

**Authors:** 


### BOOKS RECEIVED.

T. Fisher Unwin.

"Pain, its Causation and Diagnostic Significance in Internal Diseases." By Dr. Rudolph Schmidt, translated by K. M. Vogel, M.D., and H. Zinseer, M.D.

J. Wright and Sons, Ltd., Bristol.

"Manual of Natural Therapy." By Thomas D. Luke, M.D., F.R.C.S., Ed.

Baillière, Tindall, and Cox.

"Tuberculosis in Infancy and Childhood." By T. N. Kelynack, M.D.

THE

### GRADUATES' MEDICAL SCHOOLS.

DIARY FOR WEEK NOV. 2 to NOV. 6.

**NATIONAL HOSPITAL FOR THE PARALYSED AND EPILEPTIC, Queen Sq., Bloomsbury, W.C.**

**Nov. 3,** Dr. Jas. Taylor, **Syphilitic Diseases of the Spinal Cord.**

**Nov. 6,** Dr. Aldren Turner, **Neuritis.**

**THE WEST-END HOSPITAL FOR NERVOUS DISEASES, Welbeck Street, W.**

**Nov. 5,** Dr. Purves Stewart, **Myopathy.**

**ST. JOHN'S HOSPITAL FOR DISEASES OF THE SKIN, Leicester Square, W.C.**

**Nov. 5,** Chesterfield Lecture, **Seborrhœa and Psoriasis.**

**THE THROAT HOSPITAL, Golden Square, W.**

**Nov. 2,** Mr. Rose, **Rhinoliths, Foreign Bodies, Epistaxis.**

**Nov. 5,** Mr. Parker, **Chronic Catarrhal Rhinitis.**

**THE POST-GRADUATE COLLEGE, West London Hospital, Hammersmith, W.**

**At 12 noon.**

**Nov. 2,** Dr. Low, **Pathological Demonstration.**

**At 12.15 p.m.**

**Nov. 4 and 6,** Dr. Pritchard, **Practical Medicine.**

**At 5 p.m.**

**Nov. 2,** Mr. Bidwell, **Pulmonary Embolism after Laparotomy.**

**Nov. 3,** Dr. Moullin, **Gynæcology, I.**

**Nov. 4,** Dr. Beddard, **Medicine, III.**

**Nov. 5,** Mr. Dunn, **Cases of Eye Diseases.**

**Nov. 6,** Dr. Abraham, **Cases of Skin Diseases.**

**LONDON SCHOOL OF CLINICAL MEDICINE, Seamen's Hospital, Greenwich, S.E.**

**At 3.15 p.m.**

**Nov. 2,** Mr. Wm. Turner, **Some Points in Intra-cranial Surgery.**

**At 2.15 p.m.**

**Nov. 3,** Dr. Russell Wells, **Gout.**

**MOUNT VERNON HOSPITAL, Fitzroy Square, W.**

**Nov. 5,** Dr. J. E. Squires, **Hæmoptysis and its Treatment.**

**MEDICAL GRADUATES' COLLEGE AND POLYCLINIC, 22 Chenies Street, W.C.**

**Nov. 2,** Dr. A. Edmunds, **The Surgical Treatment of Tuberculous Glands.**

**Nov. 3,** Mr. V. Cargill, **Some Recent Advances in Ophthalmology.**

**Nov. 4,** Mr. G. L. Cheatle, **Chronic Mastitis and its Treatment.**

**Nov. 5,** Dr. F. J. Smith, **Some Acute Illnesses and their Management.**

**THE HOSPITAL FOR SICK CHILDREN, Great Ormond Street, W.C.**

**At 4 p.m.**

**Nov. 5,** Mr. Waugh, **The Diagnosis and Treatment of Mastoid Disease.**

### ANSWERS TO CORRESPONDENTS.

W. L. D. Child.—Five years' study at a recognised medical school and hospital is the minimum period required for every British qualification. There is no exception to this rule.

Grand Mal.—A note on this method of treatment, which originated in Germany, appeared in The Hospital of April 11, 1908, but we have heard no subsequent reports as to its value, either in mild or severe cases.

### Subscriptions.

The rate of annual subscriptions to The Hospital, either direct from the Office or through agents, is :—

| | |
|---|---|
| For the United Kingdom ... ... ... | 15s. a year |
| To the Colonies and Abroad ... ... | 17s. a year |

inclusive of postage in each case.

THE HOSPITAL October 31, 1908.

*Name* ..........................................

*Address* ..........................................

..........................................

**This Coupon must accompany manuscript or contributions intended for The Hospital.**

